# A new species of Music frog (Anura, Ranidae, *Nidirana*) from Mt Daming, Guangxi, China

**DOI:** 10.3897/zookeys.1059.68140

**Published:** 2021-09-01

**Authors:** Zhi-Tong Lyu, Zhong Huang*, Xiao-Wen Liao, Li Lin, Yong Huang, Ying-Yong Wang, Yun-Ming Mo

**Affiliations:** 1 State Key Laboratory of Biocontrol/ The Museum of Biology, School of Life Sciences, Sun Yat-sen University, Guangzhou 510275, China Sun Yat-sen University Guangzhou China; 2 Natural History Museum of Guangxi, Nanning 530012, China Natural History Museum of Guangxi Nanning China; 3 Guangxi Daming Mountain National Nature Reserve Administration, Nanning 530114, China Guangxi Daming Mountain National Nature Reserve Administration Nanning China; 4 Guangxi University of Chinese Medicine, Nanning 530200, China Guangxi University of Chinese Medicine Nanning China

**Keywords:** Bioacoustics, geography, mitochondrial DNA, morphology, nest construction

## Abstract

*Nidiranaguangxiensis***sp. nov.**, a new music frog species, is proposed, based on a series of specimens collected from Mt Daming, Guangxi, southern China. The new species is close to *N.yeae*, *N.daunchina*, *N.yaoica*, and *N.chapaensis* from southwestern and south-central China and northern Indochina, while the relationships among these species remain unresolved. *Nidiranaguangxiensis* sp. nov. can be distinguished from all known congeners by the genetic divergences in the mitochondrial 16S and COI genes, the behavior of nest construction, the advertisement call containing 6–11 rapidly repeated regular notes, and a combination of morphological characteristics. Furthermore, the *Nidirana* populations recorded in Guangxi are clarified in this work, providing valuable new information on the knowledge of the genus *Nidirana*.

## Introduction

The music frog genus *Nidirana* Dubois, 1992 was originally proposed as a subgenus of *Rana* Linnaeus, 1758. Later, *Nidirana* was controversially recognized as a full genus or a synonym of *Babina* Thompson, 1912 (Chen et al. 2005; Frost et al. 2006). Recently, comprehensive morphological, molecular, bioacoustic, and biogeographical evidence has resurrected *Nidirana* as a distinct genus (Lyu et al. 2017). The frogs of this genus usually inhabit the natural or artificial swamps, ponds, and paddy fields in the hilly regions of subtropical eastern and southeastern Asia, with some species having nest construction behavior when courting (Fei et al. 2009; Lyu et al. 2017). The known diversity of *Nidirana* increased dramatically from seven to 15 species since 2017 (Lyu et al. 2017, 2019, 2020a). Most of the newly described species were previously misidentified as other congeners, due to their conservative phenotypes (Lyu et al. 2019, 2020a, 2020b). For instance, Lyu et al. (2020b) revised multiple populations historically recorded as *Nidiranaadenopleura* (Boulenger, 1909) from China. They suggested that only the populations from Taiwan, Jiangxi, Fujian, and southern Zhejiang are the true *N.adenopleura*, and nominated some other populations as three new species: *N.guangdongensis* Lyu, Wan & Wang, 2020, *N.mangveni* Lyu, Qi & Wang, 2020, and *N.xiangica* Lyu & Wang, 2020. Lyu et al.’s (2020b) work did not clarify all historic records of *N.adenopleura*, and the taxonomic status for the records not involved in their study remains unresolved.

**Figure 1.**
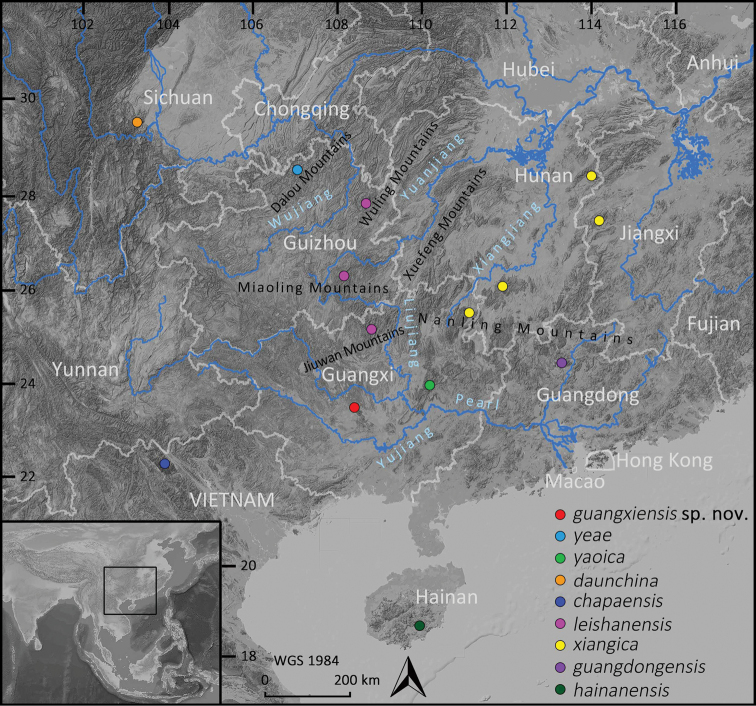
Map showing the collected localities for the *Nidirana* samples of Clade C (see Fig. 2) used in this study.

The *Nidirana* populations in Guangxi Zhuang Autonomous Region, southern China, were previously recorded as *N.adenopleura* (Liu and Hu 1962; Zhang and Wen 2000; Fei et al. 2009; Mo et al. 2014). Fei et al. (2009) suspected this identification was not correct, but still tentatively followed it and suggested additional study. Recently, the population from Mt Dayao, eastern Guangxi, has been revealed as a new species, *N.yaoica* Lyu, Mo, Wan, Li, Pang & Wang, 2019, and the population from Mt Dupangling, northeastern Guangxi was assigned to *N.xiangica* (Lyu et al. 2019, 2020b). During our recent surveys in Guangxi, we collected a series of *Nidirana* specimens from Mt Daming (**MDM**), central Guangxi, and Mt Jiuwan (**MJW**), northern Guangxi (Fig. 1). After comprehensive analyses, the specimens from MJW are identified as *N.leishanensis* Li, Wei, Xu, Cui, Fei, Jiang, Liu & Wang, 2019, while the specimens from MDM are herein proposed as a new species.

## Materials and methods

### Phylogenetic analysis

Nine muscular samples of the unnamed species from Guangxi were used for molecular analysis, encompassing five samples from MDM and four from MJW. All samples were obtained from euthanized specimens and then preserved in 95% ethanol and stored at −40 °C. In addition, 33 sequences from all known *Nidirana* species and two sequences from the outgroup, *Babinaholsti* (Boulenger, 1892) and *B.subaspera* (Barbour, 1908) (following Lyu et al. 2017), were obtained from GenBank and incorporated into our dataset. Detailed information on these materials is shown in Table 1 and Figure 1.

**Table 1.** Localities, voucher information, and GenBank numbers for all samples used in this study.

ID	Species	Locality	Voucher number	16S	COI
**1**	*Nidiranaguangxiensis* sp. nov.	China: Guangxi: Mt Daming*	NHMG 202007001	MZ677222	MZ678729
**2**	*Nidiranaguangxiensis* sp. nov.	China: Guangxi: Mt Daming*	NHMG 202007002	MZ677223	MZ678730
**3**	*Nidiranaguangxiensis* sp. nov.	China: Guangxi: Mt Daming*	NHMG 202007003	MZ677224	MZ678731
**4**	*Nidiranaguangxiensis* sp. nov.	China: Guangxi: Mt Daming*	NHMG 202007004	MZ677225	MZ678732
**5**	*Nidiranaguangxiensis* sp. nov.	China: Guangxi: Mt Daming*	NHMG 202007005	MZ677226	MZ678733
**6**	* Nidirana yaoica *	China: Guangxi: Mt Dayao*	SYS a007020	MK882276	MK895041
**7**	* Nidirana yaoica *	China: Guangxi: Mt Dayao*	SYS a007021	MK882277	MK895042
**8**	* Nidirana yaoica *	China: Guangxi: Mt Dayao*	SYS a007022	MK882278	MK895043
**9**	* Nidirana leishanensis *	China: Guangxi: Mt Jiuwan	NHMG 202007021	MZ677227	MZ678734
**10**	* Nidirana leishanensis *	China: Guangxi: Mt Jiuwan	NHMG 202007022	MZ677228	MZ678735
**11**	* Nidirana leishanensis *	China: Guangxi: Mt Jiuwan	NHMG 202007023	MZ677229	MZ678736
**12**	* Nidirana leishanensis *	China: Guangxi: Mt Jiuwan	NHMG 202007025	MZ677230	MZ678737
**13**	* Nidirana leishanensis *	China: Guizhou: Mt Leigong*	SYS a007908	MN946453	MN945209
**14**	* Nidirana leishanensis *	China: Guizhou: Mt Fanjing	SYS a007195	MN946454	MN945210
**15**	* Nidirana xiangica *	China: Guangxi: Mt Dupangling	SYS a006568	MN946442	MN945198
**16**	* Nidirana xiangica *	China: Hunan: Mt Dawei*	SYS a006492	MN946434	MN945190
**17**	* Nidirana xiangica *	China: Hunan: Mt Yangming	SYS a007273	MN946440	MN945196
**18**	* Nidirana xiangica *	China: Jiangxi: Mt Wugong	SYS a002590	MN946441	MN945197
**19**	* Nidirana yeae *	China: Guizhou: Tongzi County	CIB TZ20190608004	MN295227	MN295233
**20**	* Nidirana yeae *	China: Guizhou: Tongzi County	CIB TZ20190608005	MN295228	MN295234
**21**	* Nidirana yeae *	China: Guizhou: Tongzi County	CIB TZ20160714016	MN295231	MN295237
**22**	* Nidirana adenopleura *	China: Taiwan: Taichung City	SYS a007358	MN946445	MN945201
**23**	* Nidirana adenopleura *	China: Taiwan: Taichung City	SYS a007359	MN946446	MN945202
**24**	* Nidirana chapaensis *	Vietnam: Lao Cai: Sapa*	MNHN 2000.4850	KR827711	KR087625
**25**	* Nidirana chapaensis *	Vietnam: Lao Cai: Sapa*	MNHN 1999.5871	KR827710	/
**26**	* Nidirana daunchina *	China: Sichuan: Mt Emei*	SYS a004594	MF807822	MF807861
**27**	* Nidirana daunchina *	China: Sichuan: Mt Emei*	SYS a004595	MF807823	MF807862
**28**	* Nidirana guangdongensis *	China: Guangdong: Yingde City*	SYS a005767	MN946406	MN945162
**29**	* Nidirana guangdongensis *	China: Guangdong: Yingde City*	SYS a005768	MN946407	MN945163
**30**	* Nidirana hainanensis *	China: Hainan: Mt Diaoluo*	SYS a007669	MN946451	MN945207
**31**	* Nidirana hainanensis *	China: Hainan: Mt Diaoluo*	SYS a007670	MN946452	MN945208
**32**	* Nidirana lini *	China: Yunnan: Jiangcheng County*	SYS a003967	MF807818	MF807857
**33**	* Nidirana lini *	China: Yunnan: Jiangcheng County*	SYS a003968	MF807819	MF807858
**34**	* Nidirana mangveni *	China: Zhejiang: Mt Dapan*	SYS a006310	MN946424	MN945180
**35**	* Nidirana mangveni *	China: Zhejiang: Mt Dapan*	SYS a006311	MN946425	MN945181
**36**	* Nidirana nankunensis *	China: Guangdong: Mt Nankun*	SYS a005718	MF807839	MF807878
**37**	* Nidirana nankunensis *	China: Guangdong: Mt Nankun*	SYS a005719	MF807840	MF807879
**38**	* Nidirana occidentalis *	China: Yunnan: Mt Gaoligong*	SYS a003775	MF807816	MF807855
**39**	* Nidirana occidentalis *	China: Yunnan: Mt Gaoligong*	SYS a003776	MF807817	MF807856
**40**	* Nidirana okinavana *	Japan: Okinawa: Iriomote Island*	Not given	NC022872	NC022872
**41**	* Nidirana pleuraden *	China: Yunnan: Kunming City*	SYS a007858	MT935683	MT932858
**42**	* Nidirana pleuraden *	China: Yunnan: Wenshan City	SYS a007717	MT935671	MT932850
**43**	* Babina holsti *	Japan: Okinawa*	Not given	NC022870	NC022870
**44**	* Babina subaspera *	Japan: Kagoshima: Amami Island*	Not given	NC022871	NC022871

* Type locality.

Two mitochondrial genes, namely partial 16S ribosomal RNA gene (16S) and partial cytochrome c oxidase I gene (COI), were used for phylogenetic analysis. DNA extraction, PCR amplification, and sequencing conducted on the newly collected samples followed Lyu et al. (2019). Two gene segments, 1042 base pairs (bp) of 16S and 639 bp of COI, were concatenated seriatim into a 1681-bp matrix. The final alignment was partitioned by gene and COI was further partitioned by codon position. The partitions were tested in jmodeltest v. 2.1.2, resulting in the best-fitting nucleotide substitution models as GTR+I+G. Sequenced data were analyzed using maximum likelihood (ML) in RaxmlGUI v. 1.3 (Silvestro and Michalak 2012). The bootstrap consensus tree inferred from 1000 replicates was used to represent the evolutionary history of the taxa analyzed.

### Morphological examination

Seventeen male and two female unnamed specimens collected from MDM were examined and measured, collection information is given in the taxonomic proposal. All specimens were fixed in 10% buffered formalin, transferred to 70% ethanol, and deposited in the Natural History Museum of Guangxi (**NHMG**) and the Museum of Biology, Sun Yat-sen University (**SYS**), China.

Morphological descriptions follow the consistent definition by Lyu et al. (2017, 2019, 2020a, 2020b). External measurements of specimens were made with digital calipers (Neiko 01407A stainless steel 6-inch digital calipers) to the nearest 0.1 mm. These measurements are as follows:

**SVL** snout-vent length (from tip of snout to posterior margin of vent);

**HDL** head length (from tip of snout to the articulation of the jaw);

**HDW** head width (head width at the commissure of the jaws);

**SNT** snout length (from tip of snout to the anterior corner of the eye);

**IND** internasal distance (distance between nares);

**IOD** interorbital distance (minimum distance between upper eyelids);

**ED** eye diameter (from the anterior corner of the eye to posterior corner of the eye);

**TD** tympanum diameter (horizontal diameter of tympanum);

**TED** tympanum-eye distance (from anterior edge of tympanum to posterior corner of the eye);

**HND** hand length (from the proximal border of the outer palmar tubercle to the tip of digit III);

**RAD** radio-ulna length (from the flexed elbow to the proximal border of the outer palmar tubercle);

**FTL** foot length (from distal end of shank to the tip of digit IV);

**TIB** tibial length (from the outer surface of the flexed knee to the heel).

Sex and age were determined by examining the gonads. Webbing formula follows Savage (1975).

Comparison characters of all known congeners were obtained from129 museum specimens of 12 known congeners listed in the Appendix 1 and from the literature (Boettger 1895; Boulenger 1904, 1909; Schmidt 1925; Chang and Hsu 1932; Bourret 1937; Kuramoto 1985; Chou 1999; Fei et al. 2007, 2009; Matsui 2007; Chuaynkern et al. 2010; Lyu et al. 2017, 2019, 2020a, 2020b; Li et al. 2019; Wei et al. 2020).

Particularly, since the new *Nidirana* species from MDM is geographically and phylogenetically close to *N.yaoica*, and phylogenetically close to *N.yeae* Wei, Li, Liu, Cheng, Xu & Wang, 2020, enhanced morphometric data of these three species were used for statistical analyses in R v. 4.0.0. Due to the limited number of females collected, only male specimens were used. Data of the MDM specimens were newly measured in this work; meanwhile data of *N.yaoica* and *N.yeae* were obtained from the literature (Lyu et al. 2019; Wei et al. 2020). All measurements were ln-transformed to normalize and reduce the variance. The *t*-test was conducted with statistically similar variances (*p* > 0.05 in the Levene’s test) using car R package. Boxplots were visualized with the “ggplot2” R packages. For *t*-test and boxplots, measurements were scaled to remove allometric effects of body size in morphological analysis, using the following equation: X_a_ = X_ln_ – β ∙ (SVL_ln_ – SVL_m_), where X_a_ = adjusted value; X_ln_ = ln-transformed measurements; β = unstandardized regression coefficient for each species; SVL_ln_ = ln-transformed SVL; and SVL_m_ = overall average SVL_ln_ of all samples. Principal component analysis (PCA) was performed to reduce the dimensionality of variation in the data to find whether morphological variation form the basis of detectable group structure, using the “prcomp” function and “ggplot2” package.

### Bioacoustic analysis

Advertisement calls of the *Nidirana* population from MDM were recorded in the field at the air temperature of 18 °C by a Sony PCM D100 digital sound recorder on 20 April 2021. The recorded individuals were observed to ensure as the correct species but were not captured for conservation reasons. The sound files in wave format were sampled at 44.1 kHz with 24 bits in depth. Praat v. 6.0.27 (Boersma 2001) was used to obtain the oscillogram, sonogram, and power spectrum (window length = 0.005 s). Raven Pro v. 1.5 (Cornell Lab of Ornithology 2003–2014) was used to quantify the acoustic properties (window size = 256 points, fast Fourier transform, Hanning window with no overlap). The call duration (the time between onset of the first note and offset of the last note in a call) and call PF (peak frequency; the frequency at which max power occurs within the call) were measured for each call, and the note duration (the time between onset and offset of a note) and note interval (the time between adjacent notes in a call) were measured for each note.

## Results

### Phylogeny

**Figure 2.**
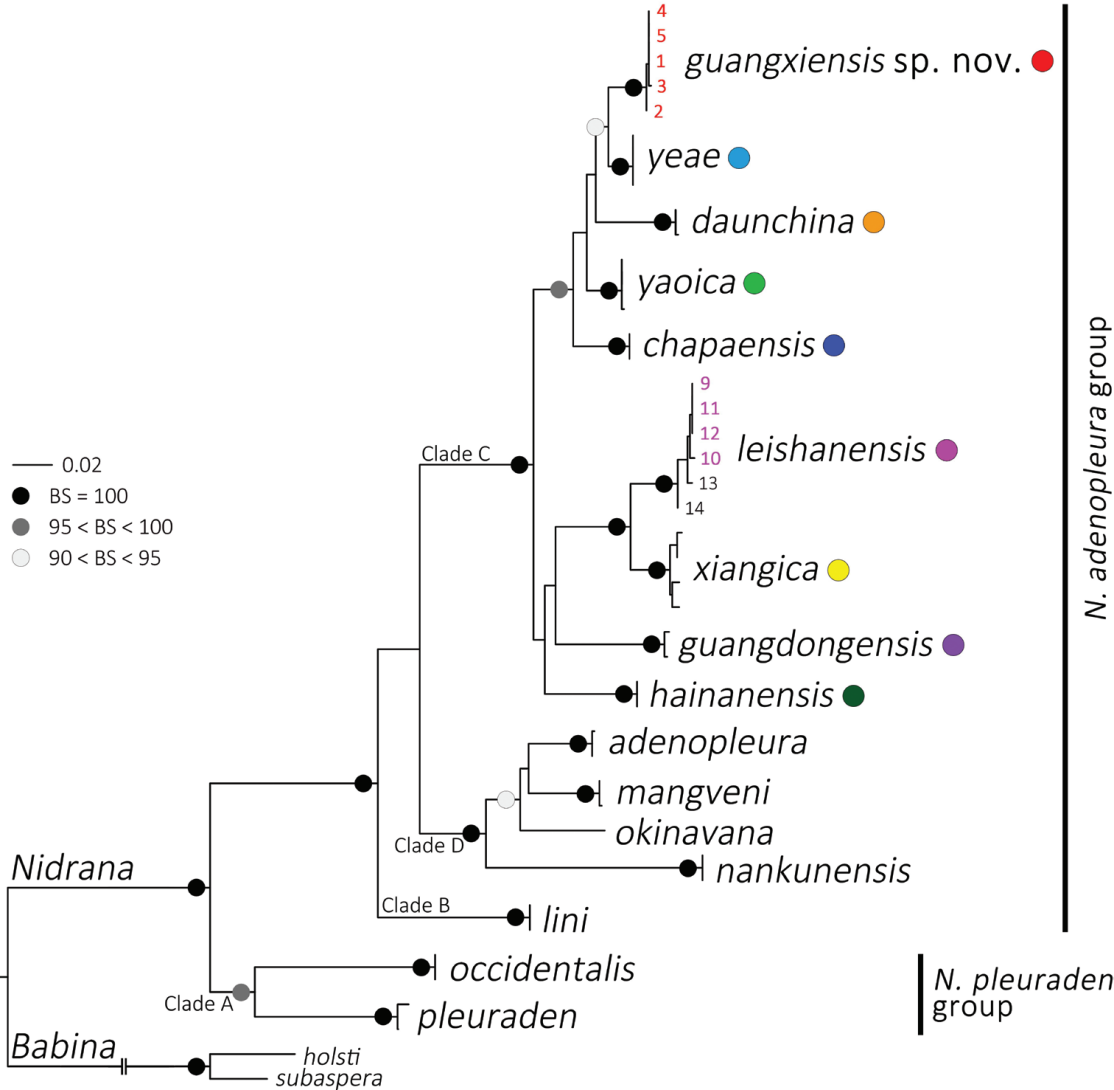
Phylogeny of *Nidirana* based on maximum likelihood. Number at the terminal of the branches corresponds to the ID number in Table 1.

The result of ML analysis was given in Figure 2, in which the supportive nodes with the bootstrap supports (BS) > 90 were shown. This mitochondrial result is consistent with the phylogenic relationship from previous studies (e.g. Lyu et al. 2020a), with two species groups and four clades revealed. The *Nidirana* populations from MDM (ID 1–5) and MJW (ID 9–12) are both inserted in the Clade C (clade names following Lyu et al. 2017) of the *N.adenopleura* group, which are distant from the true *N.adenopleura* in Clade D in phylogeny. Within Clade C, the *Nidirana* population from MJW (ID 9–12) is clustered with samples of *N.leishanensis* from Mt Leigong and Mt Fanjing, Guizhou, with strong supports (BS = 100) and small divergences, which indicates the MJW population should be clarified as *N.leishanensis*. The *Nidirana* population from MDM (ID 1–5) forms an independent lineage with strong supports (BS = 100) and almost no divergence, which is close to but diverse from the lineages of *N.yeae*, *N.daunchina* (Chang, 1933), *N.yaoica*, and *N.chapaensis* (Bourret, 1937). The relationship among these five lineages remains unresolved, even though the MDM population seems closer to *N.yeae* with medium support (BS = 92).

### Morphology

**Figure 3.**
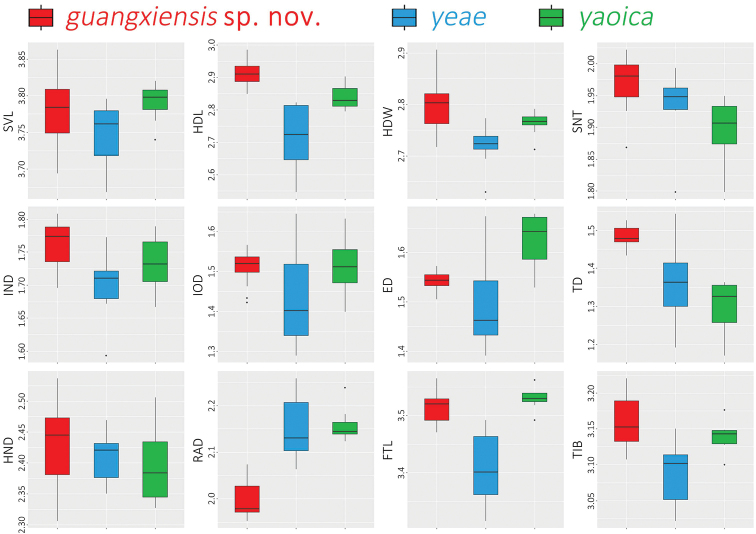
Boxplots of morphometrics based on the morphometric measurements, distinguishing *Nidiranaguangxiensis* sp. nov., *N.yeae*, and *N.yaoica*.

**Figure 4.**
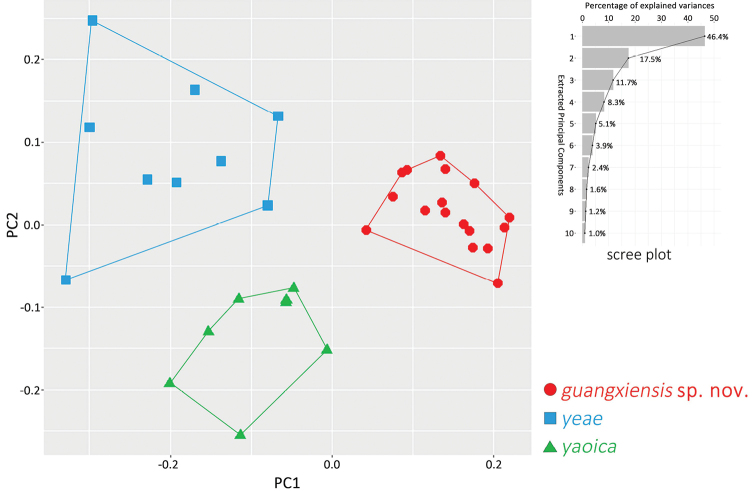
Scatter plot of PC1 and PC2 of principal component analysis based on the morphometric measurements, distinguishing *Nidiranaguangxiensis* sp. nov., *N.yeae*, and *N.yaoica*.

Detailed comparisons among all *Nidirana* species are listed in Table 2, which shows the distinct differences of the *Nidirana* specimens from MDM (detailed comparisons presented in the Taxonomic proposal below). The results of *t*-test and boxplots of morphometrics (Table 3; Fig. 3) show that the *Nidirana* specimens from MDM are significantly different from *N.yeae* from northern Guizhou, especially in the characteristics of HDL, HDW, IND, TD, RAD, FTL, and TIB, and different from *N.yaoica* from eastern Guangxi in the characteristics of HDL, HDW, SNT, ED, TD, and RAD. In the PCA analyses (Fig. 4), the extracted components PC1, PC2, PC3, and PC4 eigenvectors account for 46.4%, 17.5%, 11.7%, and 8.3% of the variance, respectively, or 83.9% cumulatively. As illustrated in the scatter plots of PC1 and PC2, samples of each species cluster together and do not overlap with each other.

**Table 2.** Diagnostic characters separating *Nidiranaguangxiensis* sp. nov. from all congeners.

Species	SVL of males (mm)	SVL of females (mm)	Fingers tips	Lateroventral groove on fingers	Relative length of fingers	Toes tips	Lateroventral groove on toes	Tibio-tarsal articulation	Subgular vocal sacs	Nuptial pad	Spinules on dorsal skin	Nest construction	Tadpole labial tooth row formula	Calling	References
* N. guangxiensis *	40.2–47.6	49.9–51.0	Dilated	Present on fingers III and IV	II < I < IV < III	Dilated	Present	Nostril	Present	One on finger I	Absent	Present	I: 1+1/1+1:II	6–11 rapidly repeated regular notes	This study
* N. yeae *	41.2–43.5	44.7	Dilated	Absent	II < IV < I < III	Dilated	Present	Eye	Present	One on finger I	Absent	? (Probably absent)	I: 1+1/1+1:II	2–6 notes containing a specific first note	Wei et al. (2020)
* N. yaoica *	42.1–45.6	?	Dilated	Present	II < I < IV < III	Dilated	Present	Nostril	Present	One on finger I	Absent	? (Probably present)	?	1–3 fast-repeated regular notes	Lyu et al. (2019)
* N. daunchina *	40.6–51.0	44.0–53.0	Dilated	Absent or rarely present	II < I < IV < III	Dilated	Present	Nostril	Present	One on finger I	Absent	Present	I:1+1/1+1:II or I:1+1/2+2:I	2–5 notes containing a specific first note	Liu (1950); Lyu et al. (2017)
* N. chapaensis *	35.5–42.5	41.0–51.8	Dilated	Present except finger I	II < I = IV < III	Dilated	Present	Nostril	Present	Two on finger I	Absent or few above vent	Present	I:1+2/1+1:II	3 notes	Chuaynkern et al. (2010)
* N. adenopleura *	43.1–57.6	47.6–60.7	Dilated	Present except finger I	II < I < IV < III	Dilated	Present	Snout tip or eye-snout	Present	One on finger I	Entire or posterior	Absent	I:1+1/1+1:II or I:0+0/1+1:I	2–5 regular notes	Lyu et al. (2017, 2020b)
* N. guangdongensis *	50.0–58.4	55.3–59.3	Dilated	Present except finger I	II < I < IV < III	Dilated	Present	Nostril	Present	One on finger I	Entire	Absent	?	2–4 regular notes	Lyu et al. (2020b)
* N. hainanensis *	32.8–44.4	?	Dilated	Present	II < I < IV < III	Dilated	Present	Nostril	Present	Absent	Absent	Present	?	2–4 fast-repeated double-notes	Fei et al. (2009, 2012)
* N. leishanensis *	49.5–56.4	43.7–55.3	Dilated	Present	II < IV < I < III	Dilated	Present	Eye-snout	Present	Two on fingers I and II	Absent	Absent	I:1+2/ 1+1:II	1 single note	Li et al. (2019)
* N. lini *	44.1–63.1	57.7–68.6	Dilated	Present except finger I	II < I < IV < III	Dilated	Present	Beyond snout	Present	One on finger I	Posterior	Absent	I:1+1/1+1:II	5–7 notes containing a specific first note	Chou (1999); Lyu et al. (2017)
* N. mangveni *	53.6–59.7	59.7–65.1	Dilated	Present on fingers III and IV	I < II < IV < III	Dilated	Present	Anterior corner of eye	Present	One on finger I	Entire or posterior	Absent	?	2–7 regular notes	Lyu et al. (2020b)
* N. nankunensis *	33.3–37.1	37.8–39.5	Dilated	Present except finger I	II < I < IV < III	Dilated	Present	Nostril	Present	One on finger I	Absent or few above vent	Present	I:1+1/1+1:II	13–15 notes containing a specific first note	Lyu et al. (2017)
* N. occidentalis *	44.5–53.0	55.6–61.3	Not dilated	Absent	II < I < IV < III	Not dilated	Absent	Eye	Present	One on finger I	Posterior	Absent	?	3–5 regular notes	Lyu et al. (2020a)
* N. okinavana *	35.5–42.8	44.6–48.8	Dilated	Present except finger I	II < I < IV < III	Dilated	Present	Eye center-near nostril	Absent	Poorly one on finger I	Absent	Present	I:1+1/1+1:II	10–25 fast-repeated notes	Chuaynkern et al. (2010); Lyu et al. (2017)
* N. pleuraden *	46.2–52.3	46.9–61.7	Not dilated	Absent	II < I < IV < III	Not dilated	Absent	Nostril	Present	One on finger I	Posterior	Absent	I:1+1/1+1: II	1–4 regular notes	Lyu et al. (2017, 2020a)
* N. xiangica *	56.3–62.3	53.5–62.6	Dilated	Present	II < I < IV < III	Dilated	Present	Eye-snout	Present	One on finger I	Entire	Absent	?	2–3 notes containing a specific first note	Lyu et al. (2020b)

**Table 3.** Morphometric comparisons based on the *t*-test of the morphometric measurements of males *Nidiranaguangxiensis* sp. nov. (*N* = 17), *N.yeae* (*N* = 9), and *N.yaoica* (*N* = 8). * *p*-values < 0.05, ** *p*-values < 0.01, *** *p*-values < 0.001.

	* guangxiensis *	* yeae *	* yaoica *	*guangxiensis vs yeae*	*guangxiensis vs yaoica*
**SVL**	40.2–47.6(43.8 ± 2.2)	39.2–44.5(42.4 ± 1.8)	42.1–45.6(44.3 ± 1.2)	0.1226	0.4136
**HDL**	17.1–19.9(18.5 ± 0.7)	12.8–16.8(15.0 ± 1.5)	16.3–18.6(17.3 ± 0.8)	0.0002 ***	0.0011 **
**HDW**	15.3–18.4(16.5 ± 0.8)	13.1–16.2(15.0 ± 0.8)	15.0–16.7(16.0 ± 0.6)	0.0002 ***	0.0232 *
**SNT**	6.4–7.8(7.2 ± 0.4)	5.7–7.3(6.8 ± 0.5)	6.2–7.2(6.7 ± 0.4)	0.1068	0.0031 **
**IND**	5.4–6.3(5.8 ± 0.2)	4.7–5.9(5.4 ± 0.3)	5.4–6.0(5.7 ± 0.2)	0.0040 **	0.1105
**IOD**	4.1–5.0(4.6 ± 0.2)	3.5–5.2(4.2 ± 0.6)	4.1–5.1(4.6 ± 0.3)	0.1730	0.8934
**ED**	4.5–4.9(4.7 ± 0.1)	4.0–5.2(4.5 ± 0.4)	4.6–5.4(5.1 ± 0.3)	0.1964	0.0068**
**TD**	4.2–4.7(4.4 ± 0.1)	3.3–4.7(3.9 ± 0.4)	3.2–3.9(3.7 ± 0.3)	0.0101*	0.0001***
**HND**	10.0–12.8(11.4 ± 0.8)	10.1–11.9(11.0 ± 0.5)	10.3–12.4(11.1 ± 0.8)	0.3092	0.2468
**RAD**	7.1–8.1(7.4 ± 0.3)	7.7–9.6(8.6 ± 0.7)	8.4–9.4(8.7 ± 0.3)	0.0001***	0.0000 ***
**FTL**	32.0–37.0(33.7 ± 1.3)	26.9–32.2(29.8 ± 1.9)	33.1–35.7(34.4 ± 0.8)	0.0006***	0.1439
**TIB**	21.9–25.2(23.7 ± 1.1)	19.6–22.8(21.5 ± 1.0)	22.6–23.9(23.3 ± 0.4)	0.0003***	0.0653

### Bioacoustics

**Figure 5.**
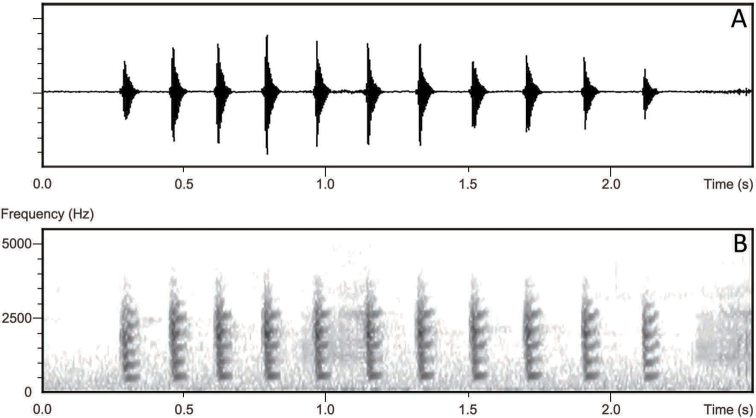
Advertisement call spectrograms of *Nidiranaguangxiensis* sp. nov. **A** waveform **B** sonogram.

The advertisement calls of three male individuals of the *Nidirana* population from MDM are recorded, and the call spectrograms are shown in Figure 5. The advertisement calls of the *Nidirana* population in MDM have the duration of 1.012–1.917 s (1.461 ± 0.29, *N* = 20), with the PF of 1894.9 Hz generally, and consisted of 6–11 (8.4 ± 1.4, *N* = 20) rapidly repeated notes. All notes are identical and regular, with the duration of 56–101 ms (77.4 ± 6.7, *N* = 168) and the interval between them lasts for 70–183 ms (110.4 ± 21.36, *N* = 147). The advertisement calls of the *Nidirana* population in MDM are different from the congeners by (1) all notes in a call are identical and regular [vs containing a significantly different first note in *N.yeae*, *N.daunchina*, *N.lini* (Chou, 1999), *N.nankunensis* Lyu, Zeng, Wang, Lin, Liu & Wang, 2017, and *N.xiangica*; containing 2–4 fast-repeated double-notes in *N.hainanensis* (Fei, Ye & Jiang, 2007)]; (2) containing 6–11 notes in a call [vs containing less than 6 notes in *N.leishanensis*, *N.chapaensis*, *N.yaoica*, *N.adenopleura*, *N.guangdongensis*, *N.occidentalis* Lyu, Yang & Wang, 2020, and *N.pleuraden* (Boulenger, 1904)].

### Taxonomic proposal

Based on the molecular, morphological, and bioacoustic differences, the population from MDM, Guangxi represents an unnamed species of genus *Nidirana* which is described here.

#### Nidirana guangxiensis

Taxon classificationAnimaliaAnuraRanidae

Mo, Lyu, Huang, Liao & Wang sp. nov.

83B21CE3-A04A-5DD1-9B9D-35B3094AA85E

http://zoobank.org/4E5C27A2-D398-4758-A181-BB49D1D5EF42

##### Chresonymy.

Hylarana (Hylarana) adenopleura – Zhang and Wen 2000 (Mt. Daming, Guangxi)

*Nidiranaadenopleura* – Mo et al. 2014 (Wuming and Shanglin, Guangxi)

##### Holotype.

**Figure 6.**
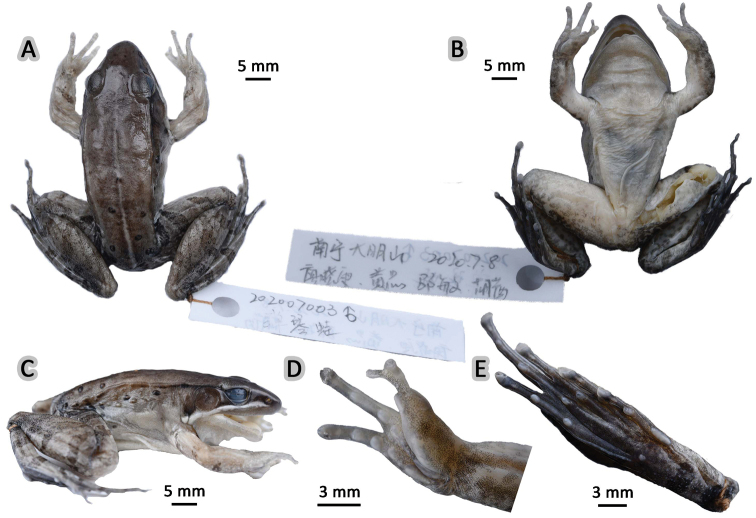
Morphological features of the adult male holotype NHMG 202007003 of *Nidiranaguangxiensis* sp. nov. in preservative **A** dorsal view **B** ventral view **C** lateral view **D** right hand **E** right foot. Photos by Shuo Qi.

**Figure 7.**
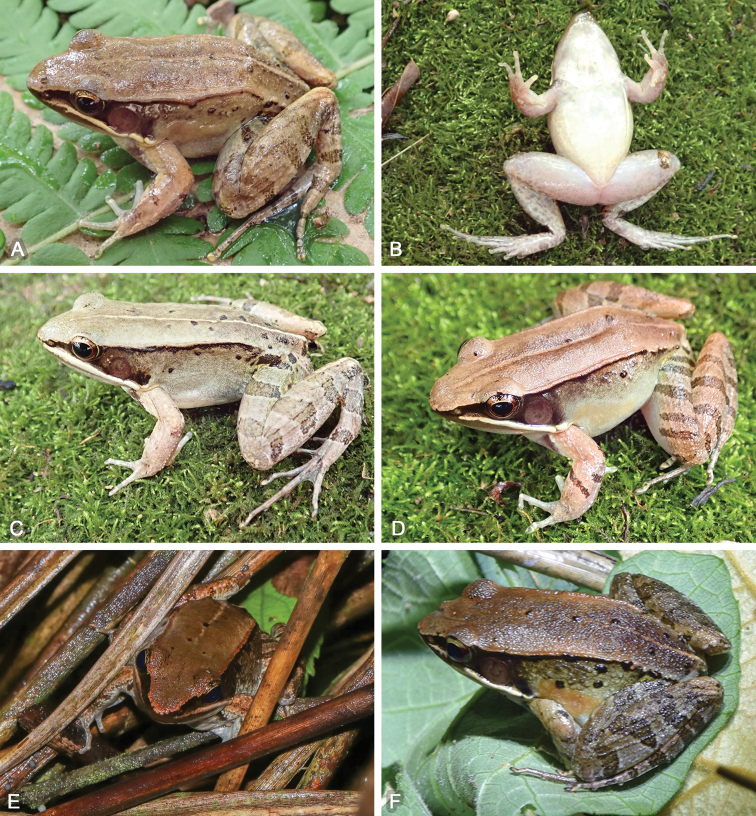
Variation and colorations of *Nidiranaguangxiensis* sp. nov. in life **A, B** male holotype NHMG 202007003 **C** female paratype NHMG 202007001 **D** male paratype NHMG 202007002 **E, F** uncaptured female and male individuals in the wild. Photos by Zhong Huang, Zhi-Tong Lyu, and Yun-Ming Mo.

NHMG 202007003 (Figs 6, 7A, B), adult male, collected by Zhong Huang and Xiao-Wen Liao on 7 July 2020 from Mt Daming (23.5156°N, 108.4370°E; ca 1260 m a.s.l.), Wuming District, Nanning City, Guangxi Zhuang Autonomous Region, China.

##### Paratypes.

Eighteen specimens. Female NHMG 202007001 (Fig. 7C), and males NHMG 202007002 (Fig. 7D), NHMG 202007004–005, 202007007–015, 202007019–020, collected at the same time with the holotype. Female SYS a008811/NHMG 202008003 and males SYS a008812–8813/NHMG 202008004–005, collected by Yun-Ming Mo, Zhong Huang, and Xiao-Wen Liao on 18 August 2020 from the same locality with the holotype.

##### Etymology.

The specific name *guangxiensis* refers to the type locality of the new species in Guangxi Zhuang Autonomous Region. The Zhuang language, one of the official languages of Guangxi Zhuang Autonomous Region, is based on the dialect of Wuming, from where the new species was collected.

##### Common name.

“Guangxi Music Frog” in English and “广西琴蛙 (guǎng xī qín wā)” in Chinese.

##### Diagnosis.

*Nidiranaguangxiensis* sp. nov. is placed in the genus *Nidirana* based on the morphological characteristics of the absence of the thumb-like structure on finger I, presence of well-developed dorsolateral folds, and the presence of suprabrachial glands in breeding males (Lyu et al. 2017). It is further assigned to the *N.adenopleura* group by the presence of lateroventral grooves on all toes (Dubois 1992; Lyu et al. 2019).

*Nidiranaguangxiensis* sp. nov. is distinguished from its congeners by the following combination of the morphological characteristics: (1) body medium sized, with SVL 40.2–47.6 mm (43.8 ± 2.2, *N* = 17) in adult males and 49.9–51.0 mm (*N* = 2) in adult females; (2) disks of digits dilated, pointed; (3) lateroventral grooves present on fingers III and IV, and each toe; (4) relative finger length II < I < IV < III; (5) lateral fringes wide on inner sides of fingers II, III and IV but absent on finger I; (6) webbing formula on toes I 2–2⅔ II 2–3 III 2½–3⅔ IV 3⅔–2½ V; (7) tibio-tarsal articulation reaching at the nostril; (8) dorsal skin rough with dense granules, several tubercles on the posterior part, flanks, and dorsal hindlimbs, without spinules on the skin; (9) distinct supernumerary tubercles below the base of fingers III and IV, palmar tubercles prominent and distinct; (10) a pair of subgular vocal sacs present; (11) a single nuptial pad on the first finger, nuptial spinules invisible; (12) suprabrachial gland large; (13) nest construction behavior present; (14) calling consisting of 6–11 rapidly repeated regular notes.

##### Comparison.

*Nidiranaguangxiensis* sp. nov. can be significantly distinguished from all other recognized congeners by the combination of the following characteristics: (1) body medium-sized, SVL 40.2–47.6 mm (*N* = 17) in adult males and 49.9–51.0 mm (*N* = 2) in adult females [vs SVL < 38 mm in adult male *N.nankunensis*; SVL > 50 mm in adult male *N.guangdongensis*, *N.mangveni*, and *N.xiangica*; SVL < 45 mm in adult female *N.yeae* and *N.nankunensis*; SVL > 53 mm in adult female *N.guangdongensis*, *N.lini*, *N.mangveni*, *N.occidentalis*, and *N.xiangica*]; (2) relative fingers length II < I < IV < III [vs II < IV < I < III in *N.yeae* and *N.leishanensis*; II < I = IV < III in *N.chapaensis*; I < II < IV < III in *N.mangveni*]; (3) lateroventral grooves present on fingers III and IV [vs absent on all fingers in *N.yeae*, *N.occidentalis*, and *N.pleuraden*; present on all fingers in *N.yaoica*, *N.hainanensis*, *N.leishanensis*, and *N.xiangica*; present on all fingers except finger I in *N.chapaensis*, *N.adenopleura*, *N.guangdongensis*, *N.lini*, *N.nankunensis*, and *N.okinavana* (Boettger, 1895)]; (4) lateroventral grooves present on all toes [vs absent on all toes in *N.occidentalis* and *N.pleuraden*]; (5) tibio-tarsal articulation reaches the nostril [vs beyond the snout tip in *N.lini*; at the eye in *N.yeae* and *N.occidentalis*]; (6) a single nuptial pad present on finger I [vs nuptial pad absent in *N.hainanensis*; nuptial pad divided into two parts on finger I in *N.chapaensis*; two nuptial pads respectively on fingers I and II in *N.leishanensis*]; (7) a pair of subgular vocal sacs present in males [vs absent in *N.okinavana*]; (8) spinules on posterior dorsal skin absent [vs present in *N.adenopleura*, *N.lini*, *N.mangveni*, *N.occidentalis*, *N.pleuraden*, and *N.xiangica*].

Particularly, *Nidiranaguangxiensis* sp. nov. is relatively close in phylogeny to *N.yeae* from northern Guizhou, but it can be distinguished by: the relative fingers length II < I < IV < III [vs II < IV < I < III in *N.yeae*]; lateroventral grooves present on fingers III and IV [vs absent on all fingers]; tibio-tarsal articulation reaches the nostril [vs at the eye]; lateral fringes wide on inner sides of fingers II, III and IV but absent on finger I [vs present only on fingers III and IV]; webbing formula on toes I 2–2⅔ II 2–3 III 2½–3⅔ IV 3⅔–2½ V [vs I 2–2 II 1⅔–3½ III 2½–3⅔ IV 3⅔–2 V]; in males, head larger, HDL/SVL 0.42 ± 0.02 [vs 0.35 ± 0.03], HDW/SVL 0.38 ± 0.02 [vs 0.35 ± 0.01], radio-ulna length shorter, RAD/SVL 0.17 ± 0.01 [vs 0.20 ± 0.01], and foot length longer, FTL/SVL 0.78 ± 0.03 [vs 0.70 ± 0.05].

##### Description of holotype.

NHMG 202007003 (Figs 6, 7A, B), adult male. Body medium-sized, SVL 43.8 mm; head relatively long and wide (HDL/SVL 0.42, HDW/SVL 0.36), longer than wide (HDW/HDL 0.86), flat above; snout rounded in dorsal and lateral views, slightly protruding beyond lower jaw, longer than horizontal diameter of eye (SNT/ED 1.57); canthus rostralis distinct, slightly curved inwards on the nostril; loreal region concave; nostril round, closer to the snout than to the eye; a longitudinal swollen mandibular ridge extending from below nostril through lower edges of eye and tympanum to above insertion of arm, where the ridge is intermittent, forming a maxillary gland and shoulder gland; supratympanic fold absent; interorbital space flat, narrower than internasal distance (IND/IOD 1.31), pineal ocellus invisible; pupil elliptical, horizontal; tympanum distinct, round, relatively large, TD/ED 0.98; vomerine ridge present, bearing small teeth; tongue cordiform, margin of the tongue notched; a pair of subgular vocal sacs present.

Forelimbs moderately robust, lower arm 0.17 of SVL and hand 0.27 of SVL; fingers thin, relative finger lengths II < I < IV < III; tip of each finger slightly dilated, forming rounded disks; lateroventral grooves on fingers III and IV, not meeting at the tip of disks; fingers free of webbing; lateral fringes present and distinct on inner and outer sides of fingers II, III and IV, but absent on finger I; subarticular tubercles prominent and rounded; supernumerary tubercles present below the base of fingers III and IV; palmar tubercles three, elliptic, large, prominent and distinct; a single nuptial pad on the dorsal surface of finger I, nuptial spinules invisible.

Hindlimbs robust, tibia 0.53 of SVL, and foot 0.76 of SVL; heels overlapping when hindlimbs flexed at right angles to axis of body; tibio-tarsal articulation reaching at the nostril when hindlimb is stretched along the side of the body; toes relatively long and thin, relative lengths I < II < V < III < IV; tip of each toe slightly dilated with remarkable elongated ventral callous pad, forming long and pointed disk; lateroventral grooves well developed on each toe, not meeting at the tip of disks; webbing moderate, formula: I 1⅓–2 II 1⅓–2⅓ III 1⅔–3 IV 3⅓–1⅓ V; lateral fringes present on inner and outer sides of each toe, forming distinct dermal flap on the lateral edges of toes I and V; subarticular tubercles rounded, prominent; inner metatarsal tubercle elliptic, length triple width; outer metatarsal tubercle indistinct, small and rounded; tarsal folds present and tarsal tubercle absent.

Dorsal skin rough with dense granules, several tubercles on the posterior part, flanks, and dorsal hindlimbs, not bearing spinules on the skin; developed dorsolateral fold from posterior margin of upper eyelid to above groin but intermittent posteriorly; a large and smooth suprabrachial gland behind base of forelimb, prominent; weak longitudinal ridges on upper arms and slightly extending to lower arm; the dorsal surfaces of thigh and tibia relatively rough with tubercles, forming several longitudinal ridges. Ventral surface of throat, body, and limbs smooth; large flattened tubercles densely arranged on the rear of thigh and around vent.

##### Color of holotype.

In life (Fig. 7A, B), dorsal surface of head and body brown; a longitudinal light brown mid-dorsal stripe faintly beginning from interorbital area, extending posteriorly to vent and become more distinct; several black spots on posterior dorsum of body; dorsolateral fold brown; upper flank brown with small black spots; lower flank light brown; suprabrachial gland yellowish brown. Dorsal forelimbs brown; dorsal hindlimbs brown, two olive crossbars on the thigh, three on the tibia, and three on the tarsus; irregular olive marks on dorsal toes. Loreal and temporal regions dark brown, tympanum pink; upper ⅓ iris brownish white and lower ⅔ iris reddish brown; maxillary gland and shoulder gland creamy white. Lips, throat, ventral surface of body and limbs creamy white; rear thigh tinged with pink and pale grey patches; ventral hand and foot pale white.

In preservative (Fig. 6), dorsal surface becomes dark brown with the mid-dorsal stripe and black spots more distinct; flank surface and the suprabrachial gland become pale; crossbars and marks on limbs dark brown; loreal and temporal regions dark brown; maxillary gland and shoulder gland more distinct; ventral surface pale grey; rear thigh and ventral foot become dark grey.

##### Variations.

Measurements of type series are given in Table 4. All specimens were similar in morphology. Females are significantly larger than males, with relatively smoother skin and fewer tubercles on dorsum and flanks. The colorations vary from pale brown to reddish brown in individuals (Fig. 7C–F). The patterns of mid-dorsal stripes are also variable but always present.

**Table 4.** Measurements (in mm) of the type series of *Nidiranaguangxiensis* sp. nov., * for the holotype, M for male, and F for female.

	NHMG 202007002	NHMG 202007003*	NHMG 202007004	NHMG 202007005	NHMG 202007007	NHMG 202007008	NHMG 202007009	NHMG 202007010	NHMG 202007011	NHMG 202007012
**Sex**	M	M	M	M	M	M	M	M	M	M
**SVL**	45.4	43.8	44.0	47.6	44.5	41.7	44.0	44.1	47.5	42.6
**HDL**	18.2	18.4	17.8	19.6	18.4	17.4	19.9	19.1	19.6	17.1
**HDW**	15.5	15.9	15.7	17.0	16.6	16.4	18.4	17.0	17.5	15.3
**SNT**	7.4	7.4	6.9	7.6	7.1	6.7	7.6	7.3	7.8	6.4
**IND**	6.0	6.0	5.7	5.9	5.6	5.7	6.0	6.1	6.3	5.4
**IOD**	4.6	4.6	4.8	5.0	4.7	4.1	4.5	4.6	4.8	4.1
**ED**	4.6	4.7	4.6	4.8	4.8	4.6	4.7	4.7	4.9	4.6
**TD**	4.4	4.6	4.2	4.6	4.4	4.3	4.4	4.3	4.7	4.3
**HND**	11.6	11.9	11.3	12.8	11.8	10.5	11.6	11.6	12.4	10.6
**RAD**	7.5	7.2	7.8	7.8	7.2	7.1	7.2	7.2	8.1	7.2
**FTL**	33.2	33.4	32.6	37.0	33.2	33.2	34.0	35.6	34.7	33.7
**TIB**	23.7	23.4	23.4	25.2	24.8	22.2	22.5	25.2	25.2	23.1
	**NHMG 202007013**	**NHMG 202007014**	**NHMG 202007015**	**NHMG 202007019**	**NHMG 202007020**	**SYS a008812 /NHMG 202008004**	**SYS a008813 /NHMG 202008005**	**NHMG 202007001**	**SYS a008811 /NHMG 202008003**	
**Sex**	M	M	M	M	M	M	M	F	F	
**SVL**	40.2	43.2	41.5	42.5	40.3	45.1	46.5	49.9	51.0	
**HDL**	18.0	18.3	18.0	18.5	18.6	18.5	18.5	20.3	20.4	
**HDW**	15.8	16.2	16.2	16.7	17.0	16.1	17.1	18.7	18.8	
**SNT**	6.7	7.3	7.2	7.3	7.0	7.4	7.5	7.9	7.8	
**IND**	5.6	5.9	5.9	5.9	5.9	5.7	5.8	6.0	6.1	
**IOD**	4.4	4.3	4.4	4.6	4.6	4.6	4.6	4.7	5.0	
**ED**	4.5	4.7	4.6	4.7	4.7	4.8	4.6	5.1	5.2	
**TD**	4.3	4.5	4.3	4.5	4.4	4.6	4.3	4.2	4.2	
**HND**	10.3	10.0	11.6	11.7	12.2	11.0	11.2	12.1	12.1	
**RAD**	7.1	7.2	7.3	7.2	7.8	7.1	7.7	8.6	8.8	
**FTL**	32.0	32.7	33.7	33.8	32.4	32.8	35.1	37.5	39.5	
**TIB**	21.9	24.3	23.6	24.7	23.2	23.0	23.7	27.1	26.7	

##### Male secondary sexual characteristics.

A pair of subgular vocal sacs, a pair of slit-like openings at posterior of jaw; a single light brown nuptial pad on the dorsal surface of finger I, nuptial spinules invisible; suprabrachial gland present.

##### Tadpole.

**Figure 8.**
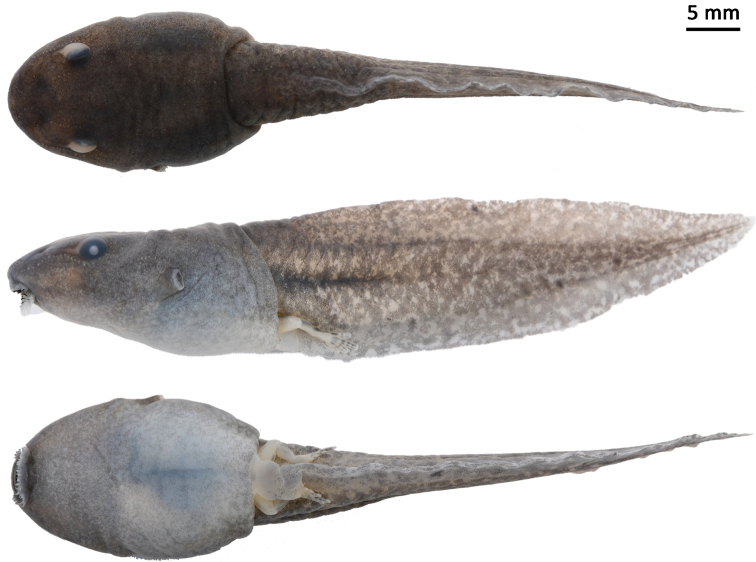
Tadpole SYS a008814 of *Nidiranaguangxiensis* sp. nov. Photos by Shuo Qi.

Body length 19.1 mm and tail length 43.1 mm in the 37^th^ stage tadpole SYS a008814 (Fig. 8); body oval, flattened above; snout rounded in dorsal aspect and profile; eyes lateral; labial tooth row formula: 1:1+1/1+1:2; spiracle on left side of body, directed dorsoposteriorly; tail depth larger than body depth; dorsal fin arising just before origin of tail, maximum depth near mid-length, tapering gradually to narrow pointed tip.

##### Distribution and ecology.

**Figure 9.**
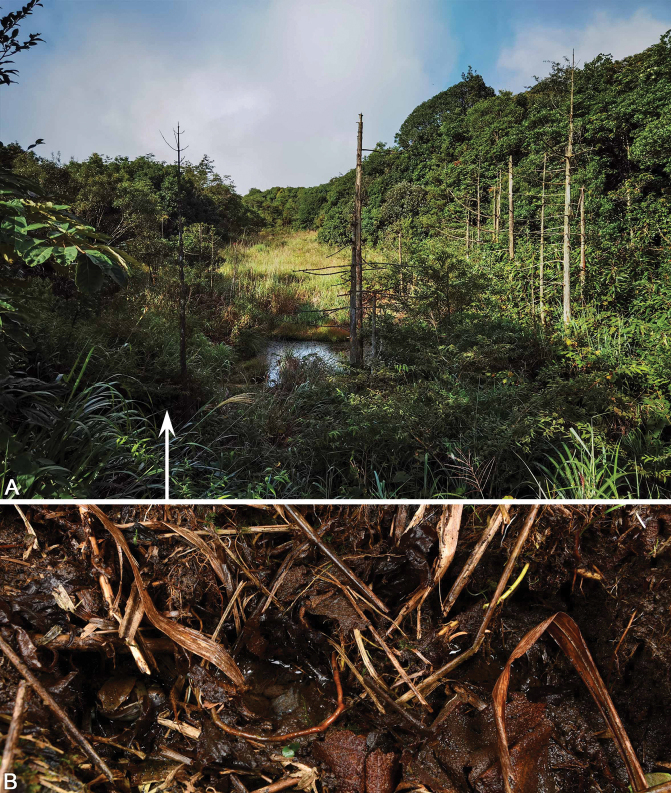
**A** habitat of *Nidiranaguangxiensis* sp. nov. in the type locality in Mt Daming **B** a male calling in a nest and two nests filled with half of water. Photos by Yun-Ming Mo and Shuo Qi.

Currently, *Nidiranaguangxiensis* sp. nov. is known only from the type locality, Mt Daming, which is located between Wuming District and Shanglin County, Nanning, Guangxi (Fig. 1). This species of frog can only be found in the alpine swamp and neighboring brushwood on the peak of Mt Daming. The estimated extent of occurrence is less than 500 km^2^, and the estimated area of occupancy is less than 50 km^2^. The swamp was surrounded by subtropical evergreen broadleaf forests (Fig. 9A). Sympatric frog species observed in the swamp are *Duttaphrynusmelanostictus* (Schneider, 1799), *Gracixalusjinxiuensis* (Hu, 1978), *Kurixalusodontotarsus* (Ye & Fei, 1993), and *Polypedatesmutus* (Smith, 1940).

*Nidiranaguangxiensis* sp. nov. was observed to have nest construction behavior. The nest is in the form of a mud burrow ca 25–30 mm in diameter and near the roots of plants. The top of the nest is open and may fill with water during the rainy season (Fig. 9B). From April to August, males call from dusk to midnight in the nest. In late April, tadpoles at the 26^th^–42^nd^ stages can be observed, with the majority at the 33^rd^–37^th^ stages.

## Discussion

With this work, the historically recorded populations of *Nidiranaadenopleura* from Guangxi, are all reassigned to other recently described species, namely *N.yaoica* from Mt Dayao in the east, *N.xiangica* from Mt Dupangling in the northeast, *N.leishanensis* from Mt Jiuwan in the north, and *Nidiranaguangxiensis* sp. nov. from Mt Daming, central Guangxi (Fig. 1). Among them, *Nidiranaguangxiensis* sp. nov. is phylogenetically close to *N.yaoica*, while *N.xiangica* and *N.leishanensis* are sister species (Fig. 2). The complex rivers and mountainous systems in Guangxi may play as important barriers to the speciation of these species pairs.

As indicated by the etymology of the generic epithet (Dubois 1992), some species of *Nidirana* were observed with the behavior of nest construction (*Nidiranaguangxiensis* sp. nov., *N.okinavana*, *N.nankunensis*, *N.hainanensis*, *N.chapaensis*, and *N.daunchina*). According to our field observations, these nest-constructing species usually live in natural swamps and ponds with muddy bottoms (Fei et al. 2009, 2012; Lyu et al. 2017; this work). Such habitats obtain seasonal rainfall and unpredictable water accumulation, which implies that constructing a nest would be helpful for the growth of the eggs and tadpoles. In contrast, the congeners without such behavior (*N.adenopleura*, *N.guangdongensis*, *N.leishanensis*, *N.lini*, *N.mangveni*, *N.occidentalis*, *N.pleuraden*, and *N.xiangica*) usually inhabit natural or artificial ponds and paddies with perennial water, which allows them to directly oviposit into the water (Fei et al. 2009, 2012; Lyu et al. 2020a, 2020b). Additionally, the nest construction behaviors of two other congeners are still unknown (*N.yaoica* and *N.yeae*; Table 2), but to roughly illustrate and compare the reported ecological data which is correlated to such courtship behavior, *N.yaoica* living in seasonal swamps (Lyu et al. 2019) is likely to construct nests, and *N.yeae* inhabiting paddy field with tadpoles observed at the water surface (Wei et al. 2020) may not possess such behavior. Regarded as important for breeding, this behavior was used for the species-group divisions (Fei et al. 2009; Chuaynkern et al. 2010). Nevertheless, Lyu et al. (2019) revised the species groups based on phylogenetic results and found that the behavior of nest construction seems to evolve independently in different clades. As an infrequent habit in the family Ranidae, the evolution of nest construction behavior in *Nidirana* species would be a topic worth studying and requires more ethological and ecological work and the application of genomic data.

Based on the phylogenetic relationships, Lyu et al. (2017) partitioned the genus *Nidirana* into four robustly supported clades (Fig. 2). Clade A corresponds to the *N.pleuraden* group with two recognized species (Lyu et al. 2019, 2020a), while the other clades belong to the *N.adenopleura* group (Lyu et al. 2019). Clade B is monotypic and includes only *N.lini*, clade D is comprised of four species, and clade C includes nine species which are more than half the members of the genus. By bringing the phylogenetic analyses from this work and previous studies (Lyu et al. 2019, 2020a, b), the interspecies relationships within clade C are unclear due to the relatively lower supported values in mitochondrial genes. Species of clade C are mostly distributed in the hilly regions throughout southwestern and south-central China and northern Indochina (Fig. 1), at the edge of the Indo-Burma biodiversity hotspot. In view of the extensiveness of these hilly areas and the unclear relationship within this clade, *Nidirana* diversity in these areas seems still underestimated, which suggests that further surveys are required.

## Supplementary Material

XML Treatment for Nidirana guangxiensis

## Appendix 1

**Specimens examined**

*Nidiranaadenopleura* (37): **China: Taiwan**: Taichung City: SYS a007358–7365; **Fujian**: Yanping District: SYS a005911–5916; Mt Wuyi: SYS a005939–5943; Jiangshi Nature Reserve: SYS a004112, 4132; Mt Yashu: SYS a005890–5891, 5901–5902; **Jiangxi**: Tongboshan Nature Reserve: SYS a001663–1665, 1667, 1698; Yangjifeng Nature Reserve: SYS a0000317, 0334; Jinggangshan Nature Reserve: SYS a004025–4027; **Zhejiang**: Jingning County: Dongkeng Town: SYS a002725–2726.

*Nidiranadaunchina* (5): **China: Sichuan**: Mt Emei: SYS a004594–4595 (topotypes); Hejiang County: Zihuai Town: SYS a004930–4932.

*Nidiranaguangdongensis* (8): **China: Guangdong**: Shimentai Nature Reserve: SYS a005765–5767, 5995, 5997–5998, 6879, 7688 (holotype and paratypes series).

*Nidiranahainanensis* (4): **China: Hainan**: Mt Diaoluo: SYS a003741, 7669–7671 (topotypes).

*Nidiranaleishanensis* (13): **China: Guizhou**: Mt Leigong: SYS a007908 (topotypes); Mt Fanjing: SYS a007195–7196; **Guangxi**: Mt Jiuwan: NHMG 202007021–023, 025–029, 042–043.

*Nidiranalini* (4): **China: Yunnan**: Jiangcheng County: Hongjiang Town: SYS a003967–3970 (topotypes).

*Nidiranamangveni* (9): **China: Zhejiang**: Mt Dapan: SYS a006310–6314; Mt. Longmen: SYS a006413–6414, 6416; Hangzhou City: SYNU 12050569 (holotype and paratypes series).

*Nidirananankunensis* (12): **China: Guangdong**: Mt Nankun: SYS a003615, 3617–3620, 4019, 4905–4907, 5717–5719 (holotype and paratypes series).

*Nidiranaoccidentalis* (8): **China: Yunnan**: Mt. Gaoligong: SYS a003775–3778; Shuangjiang County: SYS a007829–7832 (holotype and paratypes series).

*Nidiranapleuraden* (16): **China: Yunnan**: Kunming City: SYS a007585 (juvenile individual, topotype); Wenshan City: SYS a007717–7723, 7730; Xinping County: SYS a007767, 7769–7770; Shiping County: SYS a007786–7789.

*Nidiranaxiangica* (10): **China: Hunan**: Mt Dawei: SYS a006491–6493; Mt. Yangming: SYS a007269–7273; **Jiangxi**: Mt Wugong: SYS a002590–2591 (holotype and paratypes series).

*Nidiranayaoica* (13): **China: Guangxi**: Mt Dayao: SYS a007009, 7011–7014, 7020–7022, NHMG 1503043–047 (holotype and paratypes series).

## References

[B1] Boersma P (2001) Praat, a system for doing phonetics by computer. Glot International 5: 341–345.

[B2] Boettger O (1895) Neue Frösche und Schlangen von den Liukiu-Inseln. Zoologischer Anzeiger 18: 66–270.

[B3] Boulenger GA (1904) Descriptions of new frogs and snakes from Yunnan. Annals and Magazine of Natural History (Series 7) 13: 130–135. 10.1080/00222930408562447

[B4] Boulenger GA (1909) Descriptions of four new frogs and a new snake discovered by Mr. H. Sauter in Formosa. Annals and Magazine of Natural History (Series 8) 4: 492–495. 10.1080/00222930908692704

[B5] Bourret R (1937) Notes herpétologiques sur l’Indochine française. XIV. Les batraciens de la collection du Laboratoire des Sciences Naturelles de l’Université. Descriptions de quinze espèces ou variétés nouvelles. Annexe au Bulletin Général de l’Instruction Publique, Hanoi.

[B6] Chang MLY Hsu HF (1932) Study of some amphibians from Szechuan. Contributions from the Biological Laboratory of the Science Society of China, Zoological Series 8: 137–181.

[B7] Chen L Murphy RW Lathrop A Ngo A Orlov NL Ho CT Somorjai IL (2005) Taxonomic chaos in Asian ranid frogs: an initial phylogenetic resolution. The Herpetological Journal 15: 231–243.

[B8] Chou WH (1999) A new frog of the genus *Rana* (Anura: Ranidae) from China. Herpetologica 55: 389–400.

[B9] Chuaynkern Y Ohler A Inthara C Duengkae P Makchai S Salangsingha N (2010) A revision of species in the subgenus Nidirana Dubois, 1992, with special attention to the identity of specimens allocated to *Ranaadenopleura* Boulenger, 1909, and *Ranachapaensis* (Bourret, 1937) (Amphibia: Anura: Ranidae) from Thailand and Laos. Raffles Bulletin of Zoology 58: 291–310.

[B10] Dubois A (1992) Notes sur la classification des Ranidae (Amphibiens anoures). Bulletin Mensuel de la Société Linnéenne de Lyon 61: 305–352. 10.3406/linly.1992.11011

[B11] Fei L Ye CY Jiang JP (2007) A new species of Ranidae, Hylarana (Nidirana) hainanensis, from China (Amphibia: Anura). Herpetologica Sinica 11: 1–4.

[B12] Fei L Hu SQ Ye CY Huang YZ (2009) Fauna Sinica. Amphibia Vol. 3 Anura. Science Press, Beijing.

[B13] Frost DR Grant T Faivovich J Bain RH Haas A Haddad CF De Sa RO Channing A Wilkinson M Donnellan SC Raxworthy CJ Campbell JA Blotto BL Moler P Drewes RC Nussbaum RA Lynch JD Green DM Wheeler WC (2006) The amphibian tree of life. Bulletin of the American Museum of natural History 297: 1–291. 10.1206/0003-0090(2006)297[0001:TATOL]2.0.CO;2

[B14] Kuramoto M (1985) A new frog (genus *Rana*) from the Yaeyama Group of the Ryukyu Islands. Herpetologica 41: 150–158.

[B15] Li S Wei G Xu N Cui J Fei L Jiang J Liu J Wang B (2019) A new species of the Asian music frog genus *Nidirana* (Amphibia, Anura, Ranidae) from Southwestern China. PeerJ 7: e7157. 10.7717/peerj.7157 PMC6174872 30310744

[B16] Liu CC Hu SQ (1962) A herpetological report of Kwangsi. Acta Zoologica Sinica 14: 73–104.

[B17] Lyu ZT Chen Y Yang JH Zeng ZC Wang J Zhao J Wan H Pang H Wang YY (2020a) A new species of *Nidirana* from the *N.pleuraden* group (Anura, Ranidae) from western Yunnan, China. Zootaxa 4861: 43–62. 10.11646/zootaxa.4861.1.3 33055868

[B18] Lyu ZT Dai KY Li Y Wan H Liu ZY Qi S Lin SM Wang J Li YL Zeng YJ Li PP Pang H Wang YY (2020b) Comprehensive approaches reveal three cryptic species of genus *Nidirana* (Anura, Ranidae) from China. ZooKeys 914: 127–159. 10.3897/zookeys.914.36604 32132857 PMC7046709

[B19] Lyu ZT Mo YM Wan H Li YL Pang H Wang Y Y (2019) Description of a new species of music frogs (Anura, Ranidae, *Nidirana*) from Mt Dayao, southern China. ZooKeys 858: 109–126. 10.3897/zookeys.858.34363 31312093 PMC6614172

[B20] Lyu ZT Zeng ZC Wang J Lin CY Liu ZY Wang YY (2017) Resurrection of genus *Nidirana* (Anura: Ranidae) and synonymizing *N.caldwelli* with *N.adenopleura*, with description of a new species from China. Amphibia-Reptilia 38: 483–502. 10.1163/15685381-00003130

[B21] Matsui M (2007) Unmasking *Ranaokinavana* Boettger, 1895 from the Ryukyus, Japan (Amphibia: Anura: Ranidae). Zoological Science 24: 199–204. 10.2108/zsj.24.199 17409733

[B22] Mo YM Wei ZY Chen WC (2014) Colored Atlas of Guangxi Amphibians. Guangxi Science and Technology Publishing House.

[B23] Savage JM (1975) Systematics and distribution of the Mexican and Central American stream frogs related to *Eleutherodactylusrugulosus*. Copeia 2: 254–306. 10.2307/1442883

[B24] Schmidt KP (1925) New Chinese amphibians and reptiles. American Museum Novitates 175: 1–3.

[B25] Silvestro D Michalak I (2012) RaxmlGUI: a graphical front-end for RAxML. Organisms Diversity and Evolution 12: 335–337. 10.1007/s13127-011-0056-0

[B26] Wei G Li SZ Liu J Cheng YL Xu N Wang B (2020) A new species of the music frog *Nidirana* (Anura, Ranidae) from Guizhou Province, China. ZooKeys 904: 63–87. 10.3897/zookeys.904.39161 31997890 PMC6978407

[B27] Zhang YX Wen YT (2020) Amphibians in Guangxi. Guangxi Normal University Press, Guilin, China.

